# Characteristics of people who report firearm suicidal ideation in the USA

**DOI:** 10.1136/ip-2024-045341

**Published:** 2024-12-06

**Authors:** Amelia Cromwell Mueller-Williams, Mark A Ilgen, Brain M Hicks

**Affiliations:** 1 Addiction Center, Department of Psychiatry, University of Michigan Medicine, Ann Arbor, Michigan, USA; 2 Department of Psychiatry, University of Michigan Michigan Medicine, Ann Arbor, Michigan, USA

**Keywords:** Firearm, Suicide/Self-Harm, Personality, Behavior

## Abstract

**ABSTRACT:**

**Objectives:**

Firearms are the most common method of suicide, yet little is known about the attributes of people who contemplate firearm suicide. The objective of this study was to describe people who endorse firearm suicidal ideation (FASI) in terms of associations with gun ownership and experiences, mental health problems, substance use, antisocial behaviour and personality characteristics.

**Methods:**

Data were collected as part of a national online survey of adults living in the USA, the cross-sectional sample from wave 8 was analysed (N=1579). Logistic regression and analysis of variance models were fit to examine the associations between lifetime FASI and gun ownership and experience, and several mental health, substance use, antisocial behaviour and personality variables.

**Results:**

The rate of lifetime FASI was 10.2% (n=161). FASI was associated with gun ownership and more experience with firearms, as well as higher levels of depressed mood, anxiety, suicidal ideation, self-harm behaviours, past suicide attempts, alcohol and drug use, antisocial behaviour and intimate partner violence. In terms of personality traits, FASI was associated with greater negative emotions, desire for power but also feeling powerless, and lower agreeableness and conscientiousness.

**Conclusions:**

While not common, FASI is not rare and has a profile characterised by higher-intensity mental health problems, substance use, antisocial behaviour and personality traits associated with severe externalising problems and suicide. Research on FASI as a distinct construct should continue given the high lethality of firearms as a method of suicide.

**WHAT IS ALREADY KNOWN ON THIS TOPIC**

- Though most suicide deaths involve firearms, research on the psychological risk profile of people who die by firearm suicide is limited. Firearm-related risk is usually addressed using lethal means restriction, but evaluations of thoughts of using a firearm for suicide are rare in research or clinical settings. Thus, little is known about how risk factors for suicide are related to thoughts of suicide by firearm.

**WHAT THIS STUDY ADDS**

- This study may be the first report on factors associated with firearm suicidal ideation (FASI) specifically. We identify a set of characteristics that describe a profile of characteristics associated with firearm suicide ideation in a sample of adults living in the USA.

**HOW THIS STUDY MIGHT AFFECT RESEARCH, PRACTICE OR POLICY**

- The findings have implications for policymakers (eg, legislation related to firearms) and clinicians (eg, evaluating the risk of firearm suicide specifically). More research is needed to identify antecedent and temporally co-occurring risk factors associated with FASI and how they may influence firearm suicide. We suggest that an understanding of FASI should inform future intervention and prevention efforts aimed at reducing firearm suicide.

Roughly 40% of Americans live in a household with a firearm, and guns are relatively easy to acquire in the USA.^1 2^ The lethality of suicide attempts is greatly enhanced by access to firearms,^3 4^ and the majority of firearm-related deaths are suicides while firearms are involved in most suicides.^5 6^ The high lethality of firearms when used in a suicide attempt makes research on firearm suicide challenging. However, people who seriously contemplate using a firearm for suicide provide one of the best entries into understanding people who die by firearm suicide.

Factors linked to suicide likely also apply to firearm suicide. Yet, these relationships are largely untested. Firearms do not necessarily cause people to develop suicidal thoughts or behaviours but increase risk among those already vulnerable.^7^ For example, handgun ownership is associated with increased risk of firearm suicide and suicide by any method,^4^ and more experience with firearms independently elevates the risk of suicide.^8^

Despite the importance of firearm suicide, few, if any, studies have specifically evaluated thoughts of suicide using a firearm. Generally, much of the focus on firearms and suicide is on access to lethal means.^3 9^ However, knowledge is limited on who is at risk for firearm suicide. We know that most of those who die by firearm suicide are male, older and non-Hispanic white, and that risk is elevated in the context of alcohol intoxication.^10 11^ Little else is known about the characteristics associated with firearm suicidal thoughts and behaviours.

While mental health problems are the most studied predictors of suicide risk—about 90% of suicide decedents had a psychiatric disorder^12 13^—little research has investigated their link with firearm suicide. Relationships between mental health problems and firearm suicide are inconsistent, and having a psychiatric disorder may be associated with lower odds of firearm suicide versus other methods.^14^ Suicide decedents with a history of mental health treatment or a previous suicide attempt are also less likely to die by firearm.^15 16^ These findings suggest that mental health problems may be underdiagnosed or less common among people at high risk of firearm suicide.

Alcohol use is consistently associated with firearm suicide.^10 11^ Additionally, other drug use and addiction are linked to suicidal ideation and behaviours.^17^ Opioids and benzodiazepines are independently associated with suicide risk,^18 19^ with enhanced concern over concurrent use.^20 21^ Evidence is generally lacking on how these factors influence firearm suicide, except that benzodiazepines—followed by opioids—are the most commonly identified drugs among firearm suicide decedents.^22^

Firearms are also commonly used in intimate partner violence (IPV)-related suicides, suggesting that IPV is a potential firearm-specific suicide risk factor.^23^ IPV perpetration is related to a set of personality traits that are associated with suicidal ideation and attempts, including antisocial behaviour,^24 25^ aggression, impulsivity,^26 27^ neuroticism and introversion.^12^ This suggests that some personality dimensions may contribute to the association between certain behavioural outcomes and firearm suicide risk.

Considering the difficulty in studying firearm suicide deaths, firearm suicidal ideation (FASI) may be a good proxy measure that theoretically precedes firearm suicide death but has received little research focus. Improving knowledge of the correlates of FASI could meaningfully inform prevention and intervention strategies to reduce firearm suicide deaths. We aimed to describe the characteristics of people endorsing lifetime thoughts of using a firearm for suicide by examining its associations with several *potential* risk factors including gun ownership, experience with firearms, mental health problems, substance use, antisocial behaviour and personality characteristics in a sample of adults living in the USA.

## Methods

### Sample

Data were collected as part of wave 8 (5 December 2022 to 28 January 2023) of the COVID-19 Adjustment and Behaviors study,^28^ a series of online surveys conducted among US adults from June 2020 to January 2023. Recruitment was conducted by Qualtrics XM on their website and via emails to established panel members. Participants received information on the length of the survey and compensation, but specific details on survey content were not provided before opting in. Sample ascertainment used an actively managed, double-opt-in research panel designed to generate a sample consistent with age, sex and race/ethnicity characteristics of the US adult population. Qualtrics monitored data quality by verifying no non-human responses and removing duplicates or respondents who failed in-survey attention and straight-lining checks. Although these procedures help to ensure high-quality data, this is a convenience sample not a probability sample of US adults.

Of the 1582 completed surveys, 2 were removed due to illogical responses and 1 for missing FASI information. The final sample (N=1579) was 50.7% female and 49.3% male, with a mean age of 47.3 years (SD=14.8 years). The racial composition was 61.6% white, 17.6% Hispanic, 12.5% black and 8.2% other races. Most participants reported achieving a bachelor’s degree or higher (51.2%), the median income was $50 000–$74 999, and respondents most commonly resided in suburban areas (45.3%) and in the south census region (41.0%).

## Measures

### Firearm suicidal ideation

Participants rated their level of agreement on a 6-point scale from strongly disagree to strongly agree with the following item: ‘I have thought about using a gun to attempt suicide’. This variable was dichotomised to ‘yes’ (including ‘somewhat agree’, ‘agree’ and ‘strongly agree’) and ‘no’ (including ‘somewhat disagree’, ‘disagree’ and ‘strongly disagree’).

### Firearm ownership and experiences

Firearm ownership was coded based on answers to questions on owning a handgun or rifle/long gun. Firearm experience was assessed by asking ‘Do you know how to use a gun and have experience with a gun?’ (‘No’, ‘I have shot a gun a few times, but don’t know much about guns’, ‘I have shot a gun many times and know how to use common types of guns’, ‘I am fairly skilled at shooting guns, and know a lot about guns’).

### Mental health problems and substance use

Suicidal ideation, self-harm behaviour and depressed mood were assessed using the Inventory of Depression and Anxiety Symptoms (IDAS)^29^. This included responses to IDAS items on past 2-week suicidal ideation (‘I had thoughts of suicide’) and depression (‘I felt depressed’), condensing the 5-point scales (‘not at all’, ‘a little bit’, ‘moderately’, ‘quite a bit’, extremely) to ‘not at all’, ‘a little to moderately’ and ‘quite a bit or extremely*’*. A binary measure indicating any agreement with past 2-week self-harming behaviours was created based on responses to ‘I hurt myself purposefully’ and ‘I cut or burned myself on purpose’, with ‘no’ coded if the response to both questions was ‘not at all’, and ‘yes’ if the response to either question was ‘at least a little bit’ or greater. To simplify reporting of relationships between covariates and FASI, measure response categories were collapsed for descriptive purposes. The IDAS Generalised Anxiety Disorder-7 scale (α=0.94)^29^ assessed anxiety, and scores were dichotomised at the 75th percentile to indicate the potential presence of an anxiety disorder. Lifetime suicide attempt was assessed via the single item ‘Have you ever attempted suicide?’.

Substance use was measured based on past 30-day use. Alcohol use was reported as the number of binge drinking episodes (‘how many occasions have you had five or more drinks in a row?’). Nicotine use was reported as the frequency of smoking cigarettes, using smokeless tobacco, or e-cigarettes, with the highest reported frequency used. Drug use was reported as any use of prescription opioids (‘On how many occasions (if any) have you used opiates (including morphine, codeine, Demerol, Vicodin, OxyContin, Percocet’), and benzodiazepines (‘On how many occasions (if any) have you used benzodiazepines (including Librium, Valium and Xanax’).

### IPV and antisocial behaviour

If participants indicated they ever had a romantic relationship with the same partner that lasted longer than 1 month (n=1334), they completed 13 items from the Controlling and Abusive Tactics-2^30^ and Conflict Tactics Scale-2.^31^ These items report the frequency (‘never’, ‘rare’, ‘occasional’, ‘common’, ‘frequent’) of engaging in behaviours to spy on or control (eg, search partner’s purse, wallet, or cell phone; withhold car keys or disable vehicle), threaten or insult, or physically harm (eg, push, shove, slap, punch or hit, use a knife or gun) their current or most recent romantic partner (α=0.97). Antisocial behaviour was assessed using nine items that asked how many times (‘never’, ‘once or two times’, ‘several times’, ‘many times’) participants engaged in behaviours including theft, destroying property, lying and conning, aggression and violence, being arrested, and suspended or expelled from school (α=0.93).

### Personality

Feelings about power were measured by the Desire for Power scale (α=0.93), which evaluated desires to have influence, authority, power and control over others, and five items from the Feeling Powerful scale (α=0.86).^32^ The Big Five Inventory-2 short form (BFI-2-S; 30 items) was used to assess extraversion (; α=0.71), agreeableness (α=0.76), conscientiousness (α=0.78), negative emotions (α=0.85) and open mindedness (α=0.68).^33^

### Statistical analysis

Univariate logistic regression and analysis of variance models were fit to estimate the association between FASI and the categorical and continuous covariates, respectively. We also conducted multivariable analyses to determine if the associations with lifetime FASI were relatively unique and robust to strong control variables, which included sociodemographic characteristics (sex, age, race/ethnicity, income, education, census region, population density), gun experience, lifetime suicide attempt and past 2-week suicidal ideation. Lifetime suicide attempts and current suicidal ideation were included to statistically ‘control’ or adjust associations for non-specific lifetime and proximal suicide risk; gun experience was included to adjust associations for overall exposure to firearms (ie, more exposure to firearms the more likely to consider firearm suicide). Statistical significance was evaluated at α<0.005.

## Results

The prevalence of lifetime FASI was 10.2%. The sociodemographic characteristics of those who did or did not endorse FASI are shown in table 1. Those endorsing FASI were more likely to be male (64.0% vs 47.7%), younger adults ages 25–34 years (41.0% vs 24.7%), report higher income (42.9% vs 28.0% reported income ≥$100 000 per year), higher educational attainment (66.4% vs 49.6% reported a bachelor’s degree or higher), urban residence (65.8% vs 32.3%) and living in the south (53.4% vs 39.6%).

**Table 1:** Demographic characteristics by firearm suicidal ideation

	Firearm suicidal ideation				
	No n=1418		Yes n=161		χ^2^ p value
	N	%	N	%	
Sex					<0.001
Female	742	52.3	58	36.0	
Male	676	47.7	103	64.0	
Age					<0.001
18–24	39	2.8	9	5.6	
25–34	350	24.7	66	41.0	
35–44	222	15.7	56	34.8	
45–54	254	17.9	22	13.7	
55+	553	39.0	8	5.0	
Race/ethnicity					0.009
White	864	60.9	109	67.7	
Black/African American	184	13.0	14	8.7	
Other	126	8.9	4	2.5	
Hispanic	244	17.2	34	21.1	
Income					<0.001
$0–$9999	88	6.2	11	6.8	
$10 000–$24 999	151	10.6	15	9.3	
$25 000–$49 999	303	21.4	28	17.4	
$50 000–$74 999	272	19.2	23	14.3	
$75 000–$99 999	207	14.6	15	9.3	
$100 000–$149 999	227	16.0	36	22.4	
$150 000–$199 999	98	6.9	28	17.4	
$200 000+	72	5.1	5	3.1	
Education					<0.001
High school or less	249	17.6	24	14.9	
High school plus additional education	467	32.9	30	18.6	
Bachelor’s	435	30.7	53	32.9	
Master’s	208	14.7	51	31.7	
Doctorate	59	4.2	3	1.9	
Population density					<0.001
Rural	280	19.8	19	11.8	
Suburban	680	48.0	36	22.4	
Urban	457	32.3	106	65.8	
Census region					0.002
Midwest	329	23.2	21	13.0	
Northeast	252	17.8	22	13.7	
South	562	39.6	86	53.4	
West	275	19.4	32	19.9	

Table 2 shows the associations between FASI and each categorical predictor variable from omnibus tests (ORs) and adjusted logistic regression models that included control variables (adjusted ORs). For unadjusted associations, the odds of reporting FASI were almost two times higher for gun owners and four times higher for respondents with the highest level of gun experience compared with no gun experience. The likelihood of endorsing FASI was also significantly higher for those with elevated levels of mental health symptoms, including depressed mood, recent suicidal ideation, likely generalised anxiety disorder, recent self-harm and lifetime suicide attempt. A similar pattern was identified for substance use, for which all indicators were associated with increased relative odds of FASI except for binge drinking once versus never in the past 30 days. The relative odds of FASI seemed notably large for those who reported higher-intensity binge drinking, benzodiazepine use and opioid use.

**Table 2:** Associations between firearm suicidal ideation and gun ownership and experience, mental health problems and substance use

Variables	Firearm suicidal ideation									
	No n=1418	Yes n=161	OR 95% CI				Adjusted OR 95% CI*			
	N	%	OR	LB	UB	P value	OR	LB	UB	P value
Gun-related variables										
Gun owner										
No	1106	65.2	Ref.				Ref.			
Yes	312	34.8	1.89	1.34	2.68	<0.001	0.72	0.39	1.35	0.311
Gun experience										
None	744	35.6	ref			<0.001				
I have shot a gun a few times but don't know much about guns	303	16.9	1.16	0.72	1.87	0.533				
I have shot a gun many times and know how to use common types of guns	224	19.4	1.80	1.14	2.87	0.012				
I am fairly skilled at shooting guns and know a lot about guns	146	28.1	4.02	2.62	6.18	<0.001				
Mental health problems†										
Depressed mood										
None	722	11.2	Ref.			<0.001	Ref.			0.051
A little or moderately	523	32.9	4.07	2.35	7.02	<0.001	2.06	1.11	3.83	0.022
Quite a bit or extremely	164	55.9	22.01	12.91	37.54	<0.001	2.33	1.08	5.03	0.030
Generalised anxiety disorder										
Score ≤75th percentile	1409	20.5	Ref.				Ref.			
Score ≥75th percentile	161	79.5	14.16	9.46	21.19	<0.001	3.43	2.01	5.85	<0.001
Suicidal ideation										
None	1242	28.0	Ref.			<0.001				
A little to moderately	133	35.4	11.83	7.70	18.18	<0.001				
Quite a bit to extremely	34	36.6	47.89	28.58	80.26	<0.001				
Self-harm behaviour										
No	1315	31.7	Ref.			<0.001	Ref.			
Yes	103	68.3	27.54	18.68	40.59	<0.001	6.56	3.72	11.57	<0.001
Lifetime suicide attempt										
No	1332	55.9	Ref.			<0.001				
Yes	83	44.1	12.66	8.64	18.55	<0.001				
Substance use past 30 days										
Binge drinking										
Never	1094	32.9	Ref.			<0.001	Ref.			<0.001
Once	157	10.6	2.24	1.26	3.96	0.006	1.00	0.48	2.07	0.998
2–3 times	81	18.6	7.65	4.63	12.62	<0.001	3.01	1.54	5.91	0.001
1–2 times a week	42	18.6	14.74	8.56	25.39	<0.001	6.02	2.85	12.71	<0.001
At least 3 times a week	44	19.3	14.54	8.51	24.85	<0.001	2.75	1.19	6.36	0.018
Nicotine use										
Less than 1 per day	1112	1112	Ref.			<0.001	Ref.			0.323
1–5 per day	64	35.4	5.79	3.25	10.31	<0.001	1.66	0.76	3.62	0.205
Half a pack per day	83	11.8	5.17	3.01	8.88	<0.001	1.73	0.86	3.49	0.124
About a pack per day	71	13.7	4.67	2.58	8.45	<0.001	1.40	0.62	3.16	0.425
About 1.5 packs per day	78	10.6	8.00	4.90	13.07	<0.001	1.91	0.95	3.85	0.068
2 or more packs per day	10	19.9	27.31	11.6	64.16	<0.001	2.62	0.70	9.77	0.152
Benzodiazepine use										
No	1331	42.9	Ref.				Ref.			
Yes	87	57.1	20.40	13.95	29.83	<0.001	4.75	2.68	8.42	<0.001
Opioid use										
No	1322	46.0	Ref.				Ref.			
Yes	96	54.0	16.19	11.15	23.51	<0.001	3.28	1.84	5.85	<0.001

* Adjusted ORs included the covariates of sex, age, race, income, education, population density, census region, lifetime suicide attempt, past 2 week suicidal ideation and gun experience.

† Past 2 weeks, unless otherwise specified.

LB, lower bound of the 95% CI; UB, upper bound of the 95% CI.

After adjusting for the control variables, firearm ownership, depressed mood and nicotine use were no longer significantly associated with FASI. For each of these, the only control variables that remained statistically significant in the adjusted models were recent suicidal ideation, lifetime suicide attempt, age and education; additionally, population density was significant for each model except nicotine use. Anxiety, self-harm, binge drinking, benzodiazepine use and opioid use all remained significantly associated with increased odds of FASI, though the ORs were attenuated after adjusting for the control variables.

Mean T-scores for all continuous predictors of FASI and associated effect sizes are shown in table 3. Mean scores were high among those who endorsed FASI on measures of IPV (d=2.32), antisocial behaviour (mean d=1.75), desire for power (d=1.45), and negative emotions (d=0.93). Scores were significantly lower on feeling powerful (d=−1.36), extraversion (d=−0.38), agreeableness (d=−1.02), conscientiousness (d=−1.13) and open mindedness (d=−0.46). After adjusting for the control variables, the effect sizes were attenuated, with extraversion and open mindedness no longer significantly associated with FASI. The associations with IPV, antisocial behaviour, desire for power, negative emotions, low conscientiousness, and low agreeableness remained significant.

**Table 3:** Associations between firearm suicidal ideation and intimate partner violence, antisocial behaviours and personality traits

	Firearm suicidal ideation						
**Variables**	**No** **n=1418**		**Yes** **n=161**				
	**M**	**SD**	**M**	**SD**	**Cohen’s d**	**η^2^**	Partial η^2^ adjusted for covariates*
Behaviours							
Intimate partner violence	48.2	5.7	67.5	21.0	**2.32**	**0.309**	**0.074**
Antisocial behaviour	48.4	7.7	63.9	15.7	**1.75**	**0.220**	**0.049**
Personality							
Desire for power	48.7	8.7	61.9	12.5	**1.45**	**0.161**	**0.038**
Feeling powerful	51.3	9.2	38.7	9.7	**−1.36**	**0.146**	**0.031**
Extraversion	50.4	10.2	46.6	7.6	**−0.38**	**0.013**	0.002
Agreeableness	51.0	9.8	41.2	7.6	**−1.02**	**0.086**	**0.023**
Conscientiousness	51.1	9.7	40.4	7.6	**−1.13**	**0.104**	**0.021**
Negative emotions	49.1	9.8	58.1	7.5	**0.93**	**0.073**	**0.005**
Open mindedness	50.4	10.1	45.9	8.1	**−0.46**	**0.018**	0.005

Bold denotes p<0.005.

* Adjusted for sex, age, race, income, education, population density, census region, lifetime suicide attempt, past 2-week suicidal ideation and gun experience.

## Discussion

In what may be the first study of lifetime FASI, we estimated its prevalence in a survey of US adults and identified several correlates at the nexus of suicidal ideation and firearms. These analyses suggest that a set of behavioural and psychological variables could impact the likelihood of experiencing FASI, most of which were robust to strong control variables. While more research is needed to further establish temporal and causative risk factors for FASI, we identified a profile of correlates for FASI indicative of a variety of severe internalising and externalising problems including greater substance use, mental health problems, violence, lacking desired power over other people and antisocial behaviour, with corresponding lower levels of self-regulation and positive relationships with others.

Recent depressed mood and gun ownership were not significantly associated with increased odds of reporting lifetime FASI after adjusting for the control variables. The null effect of depressed mood in adjusted models is consistent with prior findings that mental health disorders and previous suicide attempts are not associated with firearm suicide deaths^15 16^ but may also be due to adjusting for recent suicidal ideation, which conceptually overlaps more with FASI. The non-significant association for gun ownership is likely due to its overlap with the more sensitive control variable ‘firearm experience’; supporting the hypothesis that having more firearm experience increases firearm suicide risk beyond simply owning a firearm.^7 8^ It is also consistent with previous findings that firearm ownership is not associated with an increased risk of suicidality.^34^

Personality traits we found were associated with FASI, are associated with specific behaviours which could be used as targets of firearm injury prevention policies. For example, extreme risk protection order laws are designed to temporarily remove firearm access from IPV perpetrators to decrease the risk of further harm to victims.^35^ Perpetrating non-fatal IPV is also associated with non-specific suicidal thoughts and behaviours,^23 36 37^ and our results indicate this relationship extends to FASI. Thus, extreme risk protection order laws could have spillover benefits for reducing firearm suicide among perpetrators of IPV. Similarly, legislation limiting firearm access for people convicted of crimes related to other antisocial behaviour may have similar effects. Overall, our findings indicate that firearm legislation focused on reducing firearm-related harm to others may also have potential benefits by reducing the risk of firearm suicide.

If FASI is a precursor to firearm suicide death, our results suggest a set of factors that could be used to increase the sensitivity of firearm-specific suicide prevention efforts. Prior research shows that enhanced anxiety symptoms, certain personality characteristics, and alcohol use are associated with an increased risk of self-harm.^38 39^ Accordingly, our findings suggest that self-harm, anxiety and binge drinking are robustly associated with lifetime FASI endorsement. Conceptualised as types of self-injurious behaviours, self-harm and substance use could reflect maladaptive coping mechanisms related to FASI. While we cannot determine if anxiety, self-harm and binge drinking precipitated FASI, these processes are among a constellation of established exacerbating risk factors.^13^ As has been identified with co-occurring alcohol use and suicidal ideation, self-injurious behaviours associated with FASI could translate into an escalating risk of death by firearm suicide.^9^ The magnification of FASI when firearms are easily accessible is especially dangerous, as firearm suicide attempts are highly lethal and associated with impulsivity.^7 40^

### Limitations

This study has several limitations. The sample is not nationally representative, and inferences are limited to this sample rather than the population of US adults. Further, we do not know the response rate and cannot weight cases (eg, on demographic variables) to align the sample with the target population. Therefore, the sample likely has unknown biases relative to the population of US adults. Furthermore, we investigated the correlates of lifetime FASI and so cannot make inferences about causal direction, which might differ for current FASI or for within-person fluctuations in FASI over time. For example, we cannot infer if any specific experiences (eg, past binge drinking) precipitated FASI or vice versa.

## Conclusion

This study provides a foundation for investigating FASI as a construct that could improve efforts to reduce firearm suicide. Additional studies are essential to further establish the convergent and discriminant validity of FASI and characterise its link to firearm suicide risk. Future work should also investigate the efficacy of firearm suicide prevention efforts focused on patterns of violent behaviour, antisocial personality, self-harm and substance use as components in risk evaluations or interventions. Many of these are established risk factors for suicide, but less is known about how they influence FASI or the risk of death by firearm suicide. Future work should also examine other factors not included here and use designs capable of determining temporal associations.

While limited, our findings suggest a constellation of factors are correlated with elevated likelihood of FASI, which may differentiate firearm-specific suicidal ideation from other methods. The associations identified support previous work showing the hazardous role of antisocial behaviours such as IPV in firearm violence, high rates of alcohol involvement among firearm suicide deaths, and that other drug use and self-harm behaviour could also play a role. Considering the high lethality of firearms as a method of suicide, FASI-specific interventions could meaningfully reduce firearm suicide deaths. Any efforts to prevent suicide by firearm should also consider what pushes individuals to act on those thoughts, and our results suggest starting points for investigating potential risk factors specific to FASI. More research on FASI and its relationship to firearm suicide could improve the efficacy of strategies to reduce firearm violence.

## Data Availability

Data are available upon reasonable request.
